# Anti-metastatic Effect of Heliotropium bacciferum on MCF-7 Human Breast Cancer Cell Line

**DOI:** 10.7759/cureus.58939

**Published:** 2024-04-24

**Authors:** Gauranga R, Vasugi Suresh, Muthamizh Selvamani, Selvaraj Jayaraman, Mohammed Asif Hussein

**Affiliations:** 1 Department of Physiology, Saveetha Dental College and Hospitals, Saveetha Institute of Medical and Technical Science (SIMATS) Saveetha University, Chennai, IND; 2 Department of Biochemistry, Saveetha Dental College and Hospitals, Saveetha Institute of Medical and Technical Science (SIMATS) Saveetha University, Chennai, IND

**Keywords:** admet, β-catenin, breast cancer cell line, anti-metastatic, mcf-7, breast cancer, anticancer, wnt, gsk3β, heliotropium bacciferum

## Abstract

Background

*Heliotropium bacciferum*, often known as wild heliotrope or wild quailplant, is a flowering plant from the borage family. This study examines the anti-metastatic impact of *H. bacciferum* on Michigan Cancer Foundation-7 (MCF-7) breast cancer cells and its ability to disrupt signaling pathways.

Aim

To explore the anti-metastatic effect of *H. bacciferum *on the MCF-7 breast cancer cell line.

Materials and methods

For this research, MCF-7 breast cancer cells were used. Cells were cultured and subjected to 3-(4,5-dimethylthiazol-2-yl)-2,5-diphenyltetrazolium bromide (MTT) assay, as well as gene expression analysis for glycogen synthase kinase 3 beta (GSK3β), wingless-related integration site 2 (Wnt2), and β-catenin. The plant extract was tested to determine if it successfully blocked the signalling pathway or not.

Results

The MTT test was performed to study the cytotoxic impact of* H. bacciferum. *At an increasing concentration of 100 μg/mL, the extract inhibited growth by 55%, whereas at 150 μg/mL, it inhibited growth by 52.5%. Maximum inhibition was seen at 150 μg/mL. *H. bacciferum* suppressed the GSK3β and Wnt2 signaling pathways in MCF-7 breast cancer cell lines, acting as an anti-metastatic and anticancer agent. The heliotrine compound in *H. bacciferum* showed high binding energy to metastatic targets such as GSK3β, Wnt2, and β-catenin. Moreover, chemical absorption, distribution, metabolism, excretion, and toxicity (ADMET) properties also support the study.

Conclusion

In this study, we can infer that *H. bacciferum* has a favourable anticancer impact on MCF-7 breast cancer cell lines and may be utilised as an anticancer drug against breast cancer cells. It can also be further evaluated for different breast cancers and cell lines.

## Introduction

Breast cancer is the most common illness worldwide and the leading cause of cancer-related deaths [1]. Breast cancer is the leading cause of mortality among women and is a potentially lethal disease. It accounts for around 23% of cancer-related fatalities in postmenopausal women and is one of the leading causes of death in this demographic. However, women continue to detect it in advanced stages because they are careless in their self-examination and clinical evaluation of their breasts; thus, it is now a global concern [2]. Cancer development includes both benign and malignant phases [3]. Because young women are now developing breast cancer, researchers are paying attention. Given the current state of knowledge, breast cancer is without a doubt the most common cause of cancer-related mortality for women under 45 [4].

Tumor-stroma ratio (TSR) plays a crucial role in assessing the tumour microenvironment and has demonstrated prognostic value in individuals diagnosed with breast, ovarian, gastric, and colorectal cancers [5]. Cancer metastasis, which is responsible for the majority of cancer-related fatalities, continues to be an enigma in terms of comprehension. The intricate movement of tumour cells toward their intended destination involves intricate interactions with various proteins and cells. By acknowledging these connections, we have made significant strides in comprehending the fundamental mechanisms that drive the mobility and adaptability of metastatic cells [6].

The field of herbal medicine involves the exploration of various phytochemicals to discover their potential medical uses. Through extensive research, it has been established that the utilisation of herbal medications can greatly benefit cancer patients by significantly improving their chances of survival in various scenarios. Recent studies have shed light on the remarkable anti-oxidative and superoxide scavenging abilities possessed by specific active components found in herbal medicine, which not only inhibit lipid peroxidation but also exhibit anti-tumour properties. Furthermore, herbal treatments exhibit beneficial effects, including antipyretic, analgesic, anti-inflammatory, and anti-cancer properties. Apart from its diverse therapeutic applications, herbal medicine is also incorporated into nutritional supplements due to its remarkable anti-inflammatory and anti-cancer effects [7].

Bioactive compounds are natural substances that exhibit distinct biological activities in the human body without providing essential nutrients. Cancer has remained the leading cause of death worldwide for many years. Studies have shown that phytochemicals sourced from various plants possess the potential to inhibit the development of cancer [8]. Herbal therapy offers a unique approach to combating cancer compared to conventional chemical medications, as it does not induce deoxyribonucleic acid (DNA) mutations in the surviving cells. Instead, natural compounds work by enhancing the immune system, inhibiting angiogenesis to halt the spread of cancer cells, detoxifying the body to prevent further accumulation of toxins, neutralizing free radicals that contribute to mutational changes leading to cancer development, and providing support to all affected organs especially those directly impacted by the disease [9]. The development of novel pharmaceuticals derived from botanical sources holds significant potential for commercial therapeutic applications [10]. Various conventional treatments including Ayurveda, Siddha, and Unani, rely on these herbal mixtures. Recently, there has been a surge in research focusing on botanicals and the remedies or compounds extracted from them utilised in traditional healing practices. This resurgence in the study of medicinal plants has prompted indigenous populations globally to turn to herbal remedies [11].

*Heliotropium bacciferum*, a notable medicinal plant belonging to the *Boraginaceae *family, has been the subject of extensive research regarding its chemical composition. Various compounds, including flavonoids, pyrrolizidine alkaloids, naphthoquinones, phenols, and terpenoids, have been identified and characterized in *Boraginaceae *plant species. These compounds have demonstrated significant pharmacological and biological effects. The biological properties of these constituents encompass anticancer, anti-inflammatory, antiviral, antiplatelet, cardiotonic, wound healing, contraceptive, and prostaglandin activities. Notably, *H. bacciferum* is recognized for its rich content of pyrrolizidine alkaloids, some of which display antihyperlipidemic, anticancer, antidiabetic, and antibacterial properties. In situations where traditional treatments have proven ineffective, alternative therapies may be the sole recourse for numerous patients in advanced stages of cancer [12].

## Materials and methods

Chemicals

The laboratory in Canada sourced trypsin-ethylenediaminetetraacetic acid (EDTA), fetal bovine serum (FBS), antibiotics-antimycotics, Dulbecco's Modified Eagle's Medium (DMEM), and phosphate-buffered saline (PBS) from Gibco (Thermo Fisher Scientific, Waltham, US). Additionally, the 5,5,6,6-tetrachloro-1,1,3,3-tetraethylbenzimidazolylcarbocyanine iodide (JC-1) and a real-time PCR kit (MESA Green) purchased from Invitrogen (Thermo Fisher Scientific, Waltham, US). It is worth noting that all chemicals and solutions used during the research were of exceptional purity and classified as analytical grade.

Extract preparation

H. bacciferum, a member of the Boraginaceae family, underwent plant extraction using the soxhlet method with methanol as the solvent. Following extraction, the resulting extract was filtered through Whatman grade 1 filter paper (Merck, St. Louis, US). The solvent was then removed under low pressure using a rotary evaporator apparatus, resulting in a thick viscous mass stored at 4°C until needed.

MCF-7 cells procurement and culture

The breast cancer cell lines known as Michigan Cancer Foundation-7 (MCF-7) were obtained from the National Center for Cell Science (NCCS) in Pune, India, and grown following the provided cell culture guidelines. These cells were nurtured in Minimum Essential Medium (MEM) supplemented with 10% FBS at 37˚C in a 5% CO_2_ atmosphere. 

Cell viability assessment by MTT

MCF-7 cells were seeded in 96-well plates at a concentration of 5 x 105 cells per well and allowed to attach overnight. Subsequently, the cells were treated with different concentrations of Heliotropium extracts in triplicates and placed in a 37˚C incubator with 5% CO_2_ for 24 hours. After the incubation period, each well was supplemented with MTT solution and further incubated at 37°C for 4 hours. The formazan crystals formed were dissolved in 200 µl of dimethyl sulfoxide (DMSO). The optical density (OD) of the resulting solution was measured at 570 nm using a spectrophotometer. This experiment was repeated three times independently to ensure reliability. The mean OD and standard deviation were calculated for each set of replicates, providing a comprehensive analysis of the results obtained.

Gene expression analysis by real-time PCR

The present material was adopted from previously reported literature [13]. The mRNA expression levels were analysed using real-time PCR, with Tri Reagent from Sigma being employed to extract total RNA. For reverse transcription, a superscript first-strand complementary DNA (cDNA) synthesis kit from Invitrogen was used according to the manufacturer's instructions, with 2 µg of total RNA from each sample being reverse transcribed. Real-time PCR was carried out using MX3000p (Stratagene, San Diego, US) utilising MESA Green PCR master mix containing SYBR green (Eurogentec, Seraing, Belgium). Melting curve analysis was conducted for primer pairs to confirm the specificity of the amplified product. Data analysis was performed using the comparative CT method, and the fold change was calculated using the 2−CT method described by Schmittgen and Livak (2008) with the assistance of CFX Manager Version 2.1 (Bio-Rad, Hercules, US).

Molecular docking

We used molecular docking to study how Heliotrine (CID: 906426) interacts with metastatic regulatory proteins like wingless-related integration site (Wnt2), glycogen synthase kinase 3 beta (GSK3β), and β-catenin. We can refer to the crystal structures of these proteins on the Protein Data Bank. For the docking process, we used a grid box measuring 90 Å × 90 Å × 90 Å with a grid spacing of 0.55 Å. We performed the docking calculations using 100 genetic algorithm cycles of the Lamarckian genetic algorithm. Finally, we visualized the results of the 3D structural complex docking using the BIOVIA Discovery Studio (Dassault Group, Paris, France).

Pharmacokinetic studies

In addition, we carried out an in-silico analysis of the pharmacokinetics of heliotrine. This analysis involved the prediction of various important parameters, such as molecular weight, topological polar surface area (TPSA), miLog P, number of rotatable bonds, and the count of hydrogen donor and acceptor atoms, based on Lipinski's criteria from 2001. To conduct this assessment, we have used the SwissADME source, which can calculate physicochemical descriptors and predict pharmacokinetic properties for small molecules [14]. Through this platform, we gained valuable insights into crucial characteristics of the drug, including its potential interactions with specific biological barriers such as the blood-brain barrier, cytochromes P450, and P-glycoproteins.

Statistical analysis

The data was expressed using the means ± SD of three separate experiments conducted in triplicate. Statistical analysis was performed using a one-way ANOVA, with results considered significant if the p-value was below 0.05.

## Results

Effect of *H. bacciferum* on MCF-7 cell viability 

**Figure 1:**
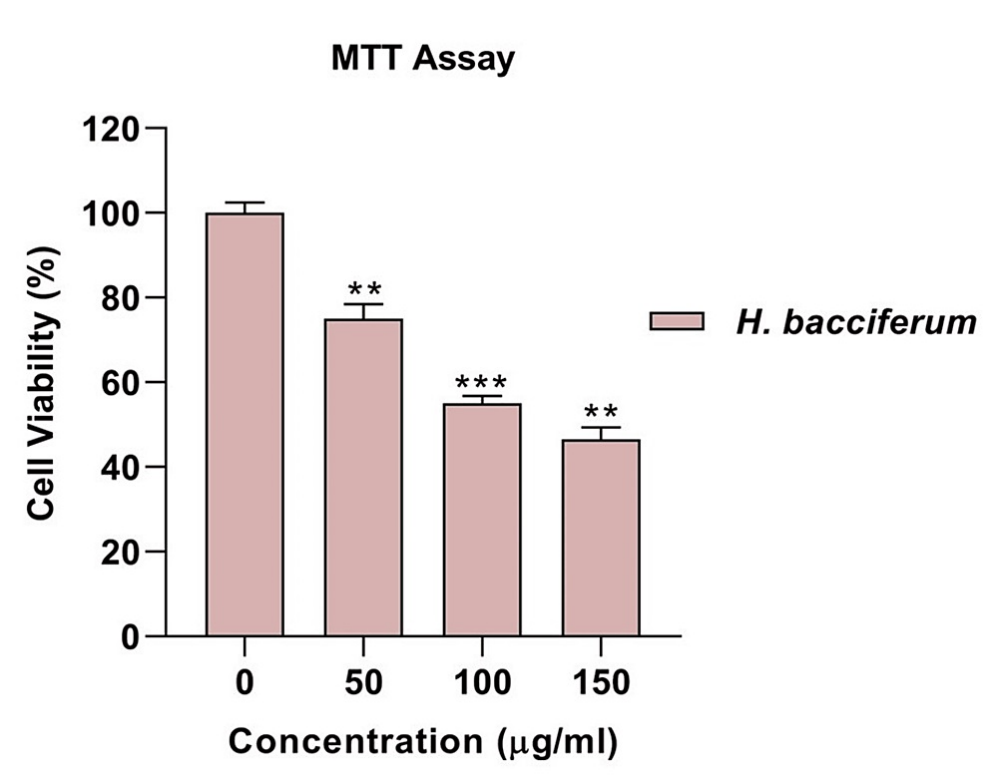
Dose-dependent cytotoxicity effect of H. bacciferum over cell viability MTT: 3-(4,5-dimethylthiazol-2-yl)-2,5-diphenyltetrazolium bromide

The current research uses an MTT assay to measure viable cell metabolism. When testing different concentrations of *H. bacciferum* on MCF-7 breast cancer cells, it was observed that H. bacciferum significantly decreased cell viability. The study demonstrated that H. bacciferum inhibited cancer cell growth in a dose-dependent manner. At 0 µg/mL concentration, there was 97.5% inhibition. At 50 µg/mL concentration, there was 75% inhibition. At 100 µg/mL concentration, there was 55% inhibition. At 150 µg/mL concentration, there was 54.5% inhibition (Figure 1).

Effects of *H. bacciferum* on mRNA expression of apoptosis signaling molecules in MCF-7 cells 

**Figure 2:**
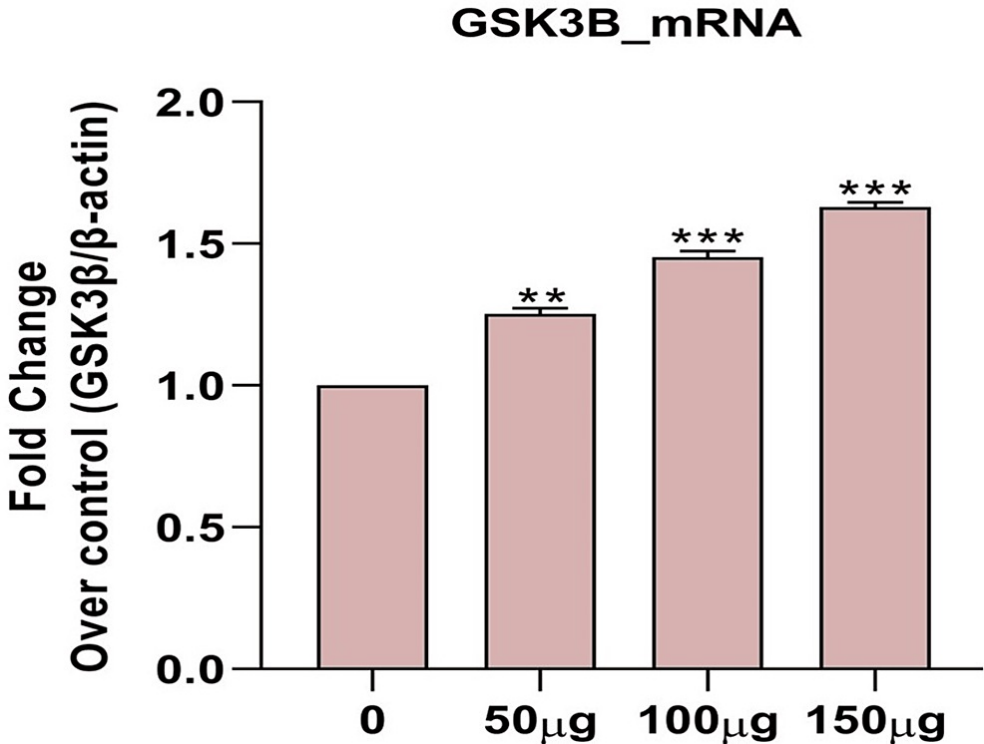
Effect of H. bacciferum (0-150 µg/ml) on GSK3β mRNA levels on MCF-7 cell lines. Each column represents mean ± SEM compared with control MCF-7 cell lines. GSK3β: Glycogen synthase kinase 3 beta; mRNA: Messenger ribonucleic acid; MCF-7: Michigan Cancer Foundation-7; SEM: Standard error of the mean

**Figure 3:**
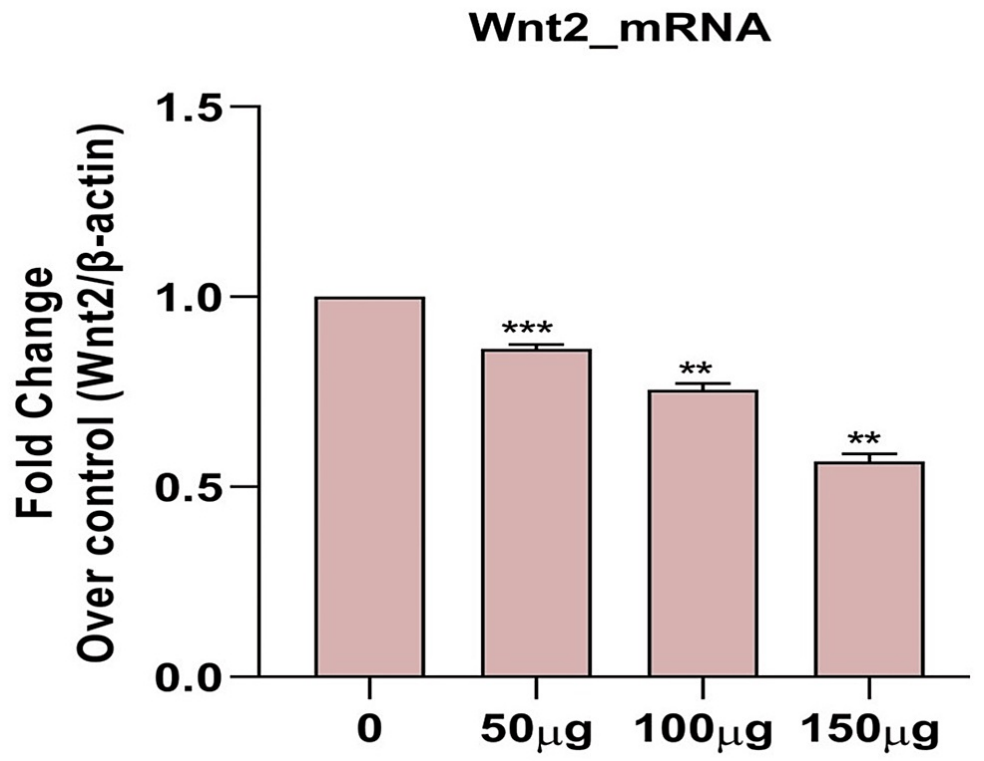
Effect of H. bacciferum (0-150 µg/ml) on WNT mRNA levels on MCF -7 cell lines. Each column represents mean ± SEM compared with control MCF-7 cell lines. WNT: Wingless-related integration site; mRNA: Messenger ribonucleic acid; MCF-7: Michigan Cancer Foundation-7; SEM: Standard error of the mean

**Figure 4:**
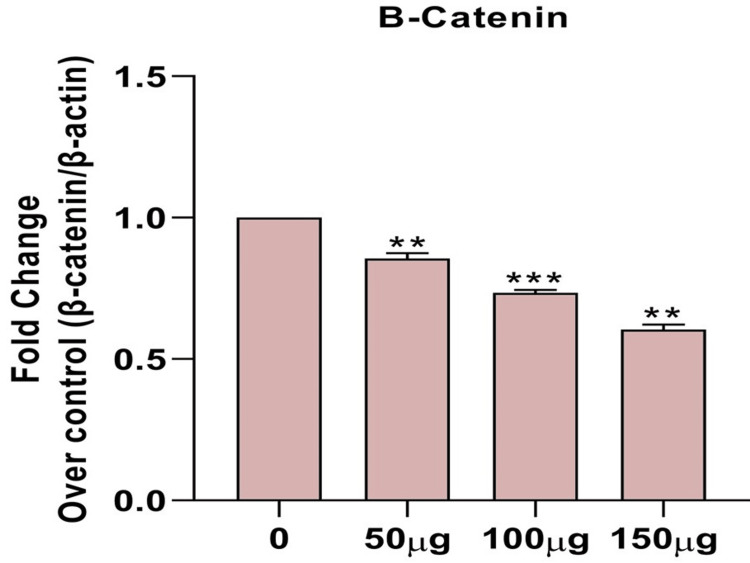
Effect of H. bacciferum (0-150 µg/ml) on B-catenin levels on MCF-7 cell lines. Each column represents mean ± SEM compared with control MCF-7 cell lines. * *mRNA: Messenger ribonucleic acid; MCF-7: Michigan Cancer Foundation-7; SEM: Standard error of the mean

H. bacciferum plant extract significantly inhibited the growth of breast cancer cells by modulating GSK3β, Wnt2, and β-catenin signaling, indicating that H. bacciferum plays a significant role in inducing breast cancer (Figures 2,3,4).

 

Molecular docking studies

**Figure 5:**
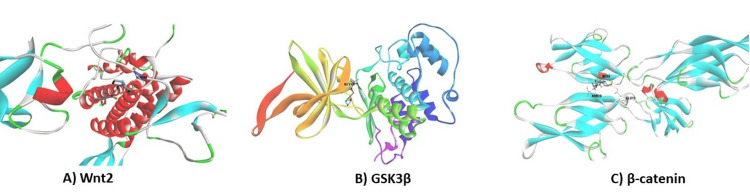
Molecular docking analysis. 3D structural illustration for heliotrine with Wnt2, GSK3β, and β-catenin complexes using BIOVIA discovery studio. A) Wnt2: Wingless-related integration site; B) GSK3: β-glycogen synthase kinase 3 beta; C) β-catenin: Beta catenin

Molecular docking was used to analyse how heliotrine interacts with proteins (Wnt2, GSK3β, and β-catenin) related to metastasis (Figure 5). Heliotrine strongly binds to GSK3β, Wnt2, and β-catenin (Table 1). The study revealed that β-catenin creates hydrogen bonds with active sites of various proteins, including Wnt2, GSK3β, and β-catenin. It suggests that heliotrine might disrupt the signaling pathway involved in metastasis, thus blocking epithelial-mesenchymal transition. These interactions demonstrate the potential of heliotrine as a cancer treatment and provide insight into the mechanisms behind its anticancer effects.

**Table 1:** Molecular docking results showing the apoptotic signaling molecules Wnt2: Wingless-related integration site; GSK3β: Glycogen synthase kinase 3 beta; β-catenin: Beta catenin

Compound	Proteins	Binding Energy (Kcal/mol)
Heliotrine	Wnt2	-6.2
	GSK3β	-6.1
	β-catenin	-5.5

 

ADME properties

In this research, we assessed heliotrine's drug-likeness using Lipinski's Rule of Five, a standard guideline for evaluating the suitability of chemical compounds as potential drugs. Heliotrine was found to have one deviation from the five criteria in Lipinski's rule, possibly due to its molecular mass, hydrogen donors, or acceptors. Table 2 illustrates these deviations graphically. Furthermore, we analysed the topological polar surface area (TPSA) of heliotrine, which plays a crucial role in determining the ability of a drug candidate to cross biological barriers such as the intestines and the blood-brain barrier. Heliotrine exhibited a high TPSA score of 79.23, indicating a preference for hydrophilicity. Although heliotrine only violated one of Lipinski's rules, suggesting a potential limitation in its oral bioavailability, its elevated TPSA value implies that its hydrophilic nature could hinder its passage across the blood-brain barrier (BBB).

**Table 2:** Heliotrine pharmacokinetic properties TPSA: Topological polar surface area; ESOL: Estimated solubility; GI: Gastrointestinal; iLOGP: Iterative log P; WLOGP: Weighted log P; MLOGP: Mean log P; MW: Molecular weight; MR: Metabolic rate

Molecule	Heliotrine
Canonical SMILES	COC(C(C(=O)OCC1=CCN2C1C(O)CC2)(C(C)C)O)C
Formula	C_16_H_27_NO_5_
MW	313.39
Heavy atoms	22
Fraction Csp3	0.81
Rotatable bonds	7
H-bond acceptors	6
H-bond donors	2
MR	85.87
TPSA	79.23
iLOGP	2.66
XLOGP3	0.91
WLOGP	-0.05
MLOGP	0.32
Silicos-IT Log P	0.86
Consensus Log P	0.94
ESOL Log S	-1.89
ESOL Class	Very soluble
Ali Log S	-2.16
Ali solubility (mol/L)	6.93E-03
Ali Class	Soluble
Silicos-IT LogSw	-0.81
Silicos-IT class	Soluble
GI absorption	High
BBB permeant	No
Pgp substrate	No
CYP1A2 inhibitor	No
CYP2C19 inhibitor	No
CYP2C9 inhibitor	No
CYP2D6 inhibitor	No
CYP3A4 inhibitor	No
log Kp (cm/s)	-7.57
Lipinski violations	0
Ghose violations	0
Veber violations	0
Egan violations	0
Muegge violations	0
Bioavailability score	0.55
PAINS alerts	0
Brenk alerts	1
Leadlikeness violations	0
Synthetic accessibility	4.75

## Discussion

The use of plants or plant-based substances is recognized for its potential in managing numerous chronic conditions, such as cancer. Research has illustrated the efficacy of various plant extracts in combating different types of cancer cells. Moreover, studies have indicated that plant extracts can induce necrosis or apoptosis in cancerous cells [15]. The MTT in vitro cell proliferation test is commonly used to assess the initial anticancer effects of natural materials, synthetic derivatives, and extracts from natural products. This dependable colorimetry-based assay can be performed with different cell lines, providing a useful evaluation of overall cell cytotoxicity. Glycogen synthase kinase-3 beta (GSK-3β) is a serine/threonine kinase that has been extensively researched for its involvement in different physiological conditions, especially cancer. Many studies indicate that this protein plays a crucial part in controlling the cell cycle [16]. Cancer development and spread greatly depend on the WNT/β-catenin signalling pathway. Targeting this pathway is crucial because it interacts with other pathways such as nuclear factor kappa B (NF-κB), Janus kinase/signal transduction and transcription activation (JAK/STAT), and Notch, giving cancer cells an advantage to survive. The activation of the WNT/β-catenin pathway is vital for cancer's survival and recurrence since it supports the maintenance of cancer stem cells, both resistant to treatment and capable of forming tumours [17].

The initial testing of *H. bacciferum*'s methanol extract did not reveal any cytotoxic effects. Nevertheless, upon increasing the dosage concentration, the extract's behaviour transitioned to exhibit cytotoxic properties. It suggests that the cytotoxicity of the *H. bacciferum* extract is contingent on the dose, with elevated concentrations resulting in cytotoxic effects [18]. When GSK-3β was introduced into MCF-7 cells, which are said to have estrogen receptors, the presence of doxorubicin caused a significant increase in the formation of colonies that rely on attachment, compared to GSK-3β wild-type (WT) or GSK-3β(A9). Regardless of whether doxorubicin was present or not, MCF-7/GSK-3β(KD) cells showed a higher efficiency in cloning compared to MCF-7/GSK-3β(WT) and MCF-7/GSK-3β(A9) cells. It is worth noting that MCF-7/GSK-3β(A9) cells produced fewer colonies than MCF-7/GSK-3β(WT) and MCF-7/GSK-3β(KD) cells. The GSK-3β(A9) gene hindered the formation of colonies, while the GSK-3β(KD) gene enhanced cloning efficiency. Colony formation is often used as an early indication of cancer development. The GSK-3β(KD) gene is likely to increase the potential for malignant transformation in MCF-7 cells, while the constitutively-active GSK-3β(A9) gene reduces it [19].

In gastric cancer cell lines OKAJIMA, TMK1, MKN7, MKN45, and MKN74, the expression of WNT3 mRNA has been observed. However, it is worth noting that WNT3A mRNA is not detected in any of the seven gastric cancer cell lines. When *Helicobacter pylori* infection occurs, the cell cycle in the gastric mucosa speeds up as a response to the damage caused by *H. pylori* virulence factors or the indirect effects of elevated levels of IFNy and TNFa. Interestingly, while TNFa significantly enhances the expression of WNT10A and WNT10B mRNA in MKN45 cells, the expression of WNT3 mRNA remains unchanged after 72 hours of treatment with TNFa or IFNy. Furthermore, WNT3A mRNA is not detectable before and after treatment with TNFa or IFNy. These findings suggest that WNT3 and WNT3A are not influenced by TNFa and IFNy signalling in MKN45 cells [20].

The development of epithelial malignancies heavily relies on the β-catenin complex. β-catenin plays a dual role in epithelial-mesenchymal transition (EMT), enhancing cell-cell adhesion at adherens junctions and acting as a coactivator for transcription once it enters the nucleus. The nuclear import of β-catenin is crucial in EMT. In MDA-MB-231 cells, β-catenin is primarily found in the nucleus, whereas in MCF-7 cells, it is predominantly expressed on the cell membrane. The presence of nuclear β-catenin in the nucleus promotes tumour invasion by increasing gene expression and transforming epithelial tumour cells into mesenchymal cells. MCF-7-14 cells exhibit β-catenin expression both on the cell membrane and the nucleus. It suggests that MCF-7-14 cells may have a higher potential for invasion due to an incomplete EMT, which alters the key signalling pathways. Various studies have identified a unique type of cell that combines epithelial and mesenchymal characteristics. These cells possess a specific metastable phenotype and differ from typical epithelial and mesenchymal cells because they retain residual E-cadherin, nuclear β-catenin, and the ability to move collectively as sheets. Based on this classification, it is reasonable to propose that MCF-7-14 cells are currently in a state of cellular metastability [21].

Limitation

The main emphasis of the study is on conducting in vitro experiments, which may not completely reflect the intricacies of the in-vivo tumor microenvironment. Moving forward, the research will expand to include in vivo studies to evaluate the impact of *H. bacciferum* on breast cancer cell lines.

## Conclusions

The anticancer properties of H. bacciferum's ethanolic extract have been demonstrated in MCF-7 cells by modulation of apoptosis signalling molecules like Wnt2, GSK3β, and β-catenin in human breast cancer cells. This research offers valuable in vitro experimental data supporting the potential of H. bacciferum as a natural therapeutic agent for breast cancer treatment. Future investigations should examine the protein expression levels of downstream signalling molecules involved in apoptosis pathways. It will provide a more comprehensive understanding of how H. bacciferum's extract interacts with these molecules and further elucidate its potential mechanisms of action in combating breast cancer. The findings of this study highlight the promising role of H. bacciferum as a potential natural drug for breast cancer therapy. Continued research into its effects on apoptosis signalling pathways and downstream molecules will contribute to developing novel treatment strategies for this prevalent and challenging disease.
